# Molecular characterization of carbapenemase-producing Enterobacterales from Peshawar City, Khyber Pakhtunkhwa, Pakistan

**DOI:** 10.1093/jacamr/dlag233

**Published:** 2026-10-23

**Authors:** Stefano Mancini, Irfan Ullah, Helena M B Seth-Smith, Natalia Kolesnik-Goldmann, Vladimira Hinic, Tim Roloff, Adrian Egli, Muhammad Ali Syed, Oliver Nolte

**Affiliations:** Institute of Medical Microbiology, University Zurich, Gloriastrasse 28/30, Zurich 8006, Switzerland; Department of Microbiology, The University of Haripur, Haripur, Pakistan; Institute of Medical Microbiology, University Zurich, Gloriastrasse 28/30, Zurich 8006, Switzerland; SIB Swiss Institute of Bioinformatics, Zurich, Switzerland; Institute of Medical Microbiology, University Zurich, Gloriastrasse 28/30, Zurich 8006, Switzerland; Institute of Medical Microbiology, University Zurich, Gloriastrasse 28/30, Zurich 8006, Switzerland; Institute of Medical Microbiology, University Zurich, Gloriastrasse 28/30, Zurich 8006, Switzerland; SIB Swiss Institute of Bioinformatics, Zurich, Switzerland; Institute of Medical Microbiology, University Zurich, Gloriastrasse 28/30, Zurich 8006, Switzerland; Department of Microbiology, The University of Haripur, Haripur, Pakistan; Institute of Medical Microbiology, University Zurich, Gloriastrasse 28/30, Zurich 8006, Switzerland

## Abstract

**Background/Objectives:**

The dissemination of multidrug-resistant Enterobacterales poses a critical public health challenge, particularly in resource-limited regions with high antimicrobial use and limited surveillance. We analysed the phenotypic and genotypic resistance profiles of carbapenemase-producing Enterobacterales collected in 2023 from three tertiary care hospitals in Peshawar, Pakistan.

**Methods:**

We performed antimicrobial susceptibility testing on 32 Enterobacterales isolates suspected of carbapenemase production using disk diffusion, gradient diffusion, and broth microdilution according to EUCAST guidelines. We conducted whole-genome sequencing to identify resistance genes, sequence types, and mutations associated with reduced susceptibility to novel β-lactams.

**Results:**

The collection comprised 16 *Escherichia coli*, 13 *Klebsiella pneumoniae*, and 3 *Proteus mirabilis* isolates. The predominant carbapenemase gene was *bla*_NDM-5_ (90.6%), frequently combined with *bla*_OXA-48-like_ variants (46.9%). ESBL genes, mainly *bla*_CTX-M-15_, were common (78.1%), while CMY-type AmpC β-lactamases were detected in 28.1% of isolates. Insertions in the PBP3-encoding gene *ftsI* and CMY co-production were associated with elevated aztreonam–avibactam MICs in *E.coli*. Reduced cefiderocol susceptibility was observed mainly among NDM-producing *E. coli* ST2 isolates carrying *cirA* disruption and *ftsI* insertions. All isolates remained susceptible to colistin, most of them to gentamicin (84.4%), and exhibited wild-type MIC-distributions for tigecycline, and eravacycline. Genomic analysis identified clustering of high-risk clones, suggesting ongoing local transmission. Notably, 11/13 *K. pneumoniae* ST147 isolates carried multiple virulence-associated genes consistent with hypervirulence.

**Conclusions:**

High-risk NDM-producing Enterobacterales with increasingly complex resistance and virulence mechanisms are circulating in Pakistan, including determinants compromising last-line β-lactams. Strengthened genomic surveillance is essential to guide therapy and limit further spread.

## Introduction

Multidrug-resistant (MDR) Enterobacterales, particularly those producing carbapenemases and extended-spectrum β-lactamases (ESBLs), represent a serious clinical concern worldwide. South Asia, including Pakistan, is a known hotspot for such resistance determinants, particularly NDM- and OXA-type carbapenemases, which frequently circulate within high-risk pandemic lineages such as *Klebsiella pneumoniae* ST147.^1–3^ Understanding the molecular epidemiology of resistance mechanisms in local clinical isolates is critical to guide empirical therapy and infection control strategies.

We aimed to characterize the antimicrobial susceptibility profiles and underlying resistance mechanisms carbapenemase-producing Enterobacterales (CPE) recovered from clinical isolates collected during 2023 from the Khyber Teaching Hospital, the Northwest General Hospital and Research Centre, and the Rehman Medical Institute, all located in Peshawar City, Khyber Pakhtunkhwa, Pakistan. We systematically investigated major β-lactam resistance mechanisms, including carbapenemases, ESBLs, and AmpC-type β-lactamases, along with 16S rRNA methylases conferring pan-aminoglycoside resistance, and plasmid-mediated colistin resistance (*mcr* genes), as well as susceptibility to conventional and second-line antibiotics.

## Materials and methods

### Ethics statement

The study was conducted in accordance with the Declaration of Helsinki and was approved by the Advance Studies and Research Board (ASRB) of The University of Haripur after technical, ethical and safety consideration (F22-0046-MIC-M.PHIL/UOH). Because the isolates were obtained from routine diagnostic samples and patient identity was strictly kept confidential, the requirement for informed consent was waived by the ethics committee. However, relevant demographic data (age, gender, sampling date, and specimen type) were recorded for each isolate.

### Sample collection and identification

Enterobacterales clinical strains suspected of carbapenemase production based on EUCAST criteria,^4^ collected during the period from July to December, 2023, at the Khyber Teaching Hospital, the Northwest General Hospital and the Research Centre, and Rehman Medical Institute following consecutive convenience sampling. All isolates were from Peshawar City, Khyber Pakhtunkhwa, Pakistan and were subjected to further analysis. The clinical samples comprised of urine, blood cultures, pus swabs, wound swabs, and sputum (Table S1, available as Supplementary data at *JAC-AMR* Online). We obtained patient demographic data at the time of sample collection. We performed initial species identification using standard biochemical methods and the API-20E system (bioMérieux, La Balme-les-Grottes, France). We assessed antimicrobial susceptibility using the Kirby-Bauer disk diffusion (DD) method, following EUCAST guidelines.^5^ Strains were subsequently shipped to the Institute of Medical Microbiology (IMM), University of Zurich, for further genotypic and phenotypic characterization. At the IMM, we confirmed species identification using MALDI-TOF mass spectrometry (Bruker Sirius, Bremen, Germany), and additional phenotypic analyses were performed as described below.

### Antimicrobial susceptibility testing

We tested all isolates for antimicrobial susceptibility at the IMM using a panel of commonly prescribed antibiotics. We employed the Kirby-Bauer DD method to assess susceptibility to classical β-lactams (piperacillin–tazobactam, ceftazidime, cefepime, meropenem, and imipenem), aminoglycosides (amikacin, gentamicin, and tobramycin), and fluoroquinolones (ciprofloxacin and levofloxacin). We used Mueller–Hinton agar plates (bioMérieux, Marcy L’Etoile, France), and inhibition zones were measured digitally using the SIRweb/SIRscan system (i2a).^6^ We tested second-line agents including aztreonam–avibactam, tigecycline, and eravacycline using gradient diffusion strips (Liofilchem, Roseto degli Abruzzi, Italy). We read the minimum inhibitory concentrations (MICs) after 18 hours of incubation at 35 °C. For colistin and cefiderocol we performed broth microdilution (BMD) using UMIC^®^ (Bruker, Germany) and ComASP^®^ Cefiderocol panels (Liofilchem), respectively, following the manufacturers’ instructions. Results were interpreted using EUCAST clinical breakpoints.^7^

### Whole genome sequencing (WGS)

We performed WGS at the IMM, University of Zurich, under ISO 17025 accreditation. We extracted genomic DNA using either the DNeasy^®^ UltraClean^®^ Microbial Kit (Qiagen, Hilden, Germany) or the Maxwell^®^ RSC Genomic DNA Kit (Promega, Madison, WI, USA), following the manufacturers’ instructions. We performed library preparation using the QIASeq FX DNA Library Kit (Qiagen) and conducted paired-end sequencing (2 × 150 bp) on Illumina MiSeq or NextSeq1000 platforms (Illumina^®^, San Diego, CA, USA), ensuring a minimum average read depth of 30×. We performed multilocus sequence typing (MLST) using Ridom SeqSphere+ v10.5.4 using established schemes (cgMLST.org). The cluster thresholds used are the published standards: 10 for *E. coli* and *P. mirabilis* and 15 for *K. pneumoniae*. We used the IMMense bioinformatic pipeline for standard analysis. In addition, we extracted nucleotide sequences of genes suspected as being involved in cefiderocol resistance (*cirA, efeO, exbB, exbD, fecA, fiu, tonB*) from Bakta v1.9.3^8^ annotated genomes and aligned and visualized in Aliview v1.27.^9^ We identified virulence-associated genes using Kleborate v3.1.3, and virulence scores were assigned as follows: 0, absence of yersiniabactin, colibactin, and aerobactin; 1, yersiniabactin only; 2, yersiniabactin and colibactin (or colibactin alone); 3, aerobactin without yersiniabactin or colibactin; 4, aerobactin with yersiniabactin (without colibactin); and 5, presence of yersiniabactin, colibactin, and aerobactin.^10^

### Detection of β-lactam resistance genes

We assembled raw sequencing reads using the IMMense pipeline (https://gitlab.uzh.ch/appliedmicrobiologyresearch/immense), including identification of plasmid-mediated and chromosomal β-lactamase genes using NCBI AMRFinderPlus v3.12.8.^11^ We deposited all genome sequences in the European Nucleotide Archive (ENA) under project number PRJEB105403 (Table S1).

## Results

### Detection of resistance mechanisms

We detected carbapenemase genes in all 32 Enterobacterales clinical strains, including *Escherichia coli* (*n* = 16), *Klebsiella pneumoniae* (*n* = 13), and *Proteus mirabilis* (*n* = 3, see Table S1). The most prevalent carbapenemase gene was *bla*_NDM-5_, detected in 29 isolates (90.6%). *bla*_NDM-5_ occurred alone in 14 isolates and in combination with *bla*_OXA-232_ in another 14 isolates. One isolate co-harboured *bla*_NDM-5_ and *bla*_OXA-484_. All three *P. mirabilis* isolates carried *bla*_NDM-1_. An ESBL gene of the *bla*_CTX-M-15_ type was identified in 22 isolates. AmpC-type β-lactamase genes were found in nine isolates: *bla*_CMY-145_ (*n* = 5), *bla*_CMY-42_ (*n* = 2), and co-carriage of *bla*_CTX-M-15_ with *bla*_CMY-2_ in two isolates.

The most frequently detected aminoglycoside-modifying enzyme (AME) gene was *aac(6’)-Ib*, which is associated with reduced susceptibility or resistance to amikacin and tobramycin. *Aac(6’)-Ib* was present in 17 of 32 (53%) isolates, either alone or in combination with other AME genes or rRNA methyltransferases (RMTases). We identified RMTases in three isolates: *armA* in one NDM-5–producing *E. coli* strain and *rmtB* in two NDM-5/OXA-232–producing strains. We did not detect plasmid-mediated colistin resistance mechanism in any of the CPE isolates.

### Clonal relatedness

The *E. coli* isolates exhibited several sequence types (STs, Figure 1a), with ST2 being the most abundant (*n* = 8). Three clusters within ST2 comprised each two to three isolates and were recovered from urine and wound swabs from seven different patients treated in three different hospital centres located in the same city (Khyber Teaching Hospital, Rehman Medical Institute, Northwest General Hospital). This clustering pattern suggests the occurrence of possible intra- and inter-hospital transmission events. Three NDM-5–producing *E. coli* isolates belong to a single ST650 cluster; we collected these isolates from surgical and wound swabs from three patients treated in two centres (Khyber Teaching Hospital, Rehman Medical Institute). Two additional NDM-5–producing *E. coli* ST664 isolates formed one cluster differing by only one allele in cgMLST and were recovered from pus swabs from two patients at Khyber Teaching Hospital. One isolate each of ST635 and ST1000 was detected, and one distantly related *E. coli* isolate carried a novel Pasteur-based ST.

**Figure 1.**
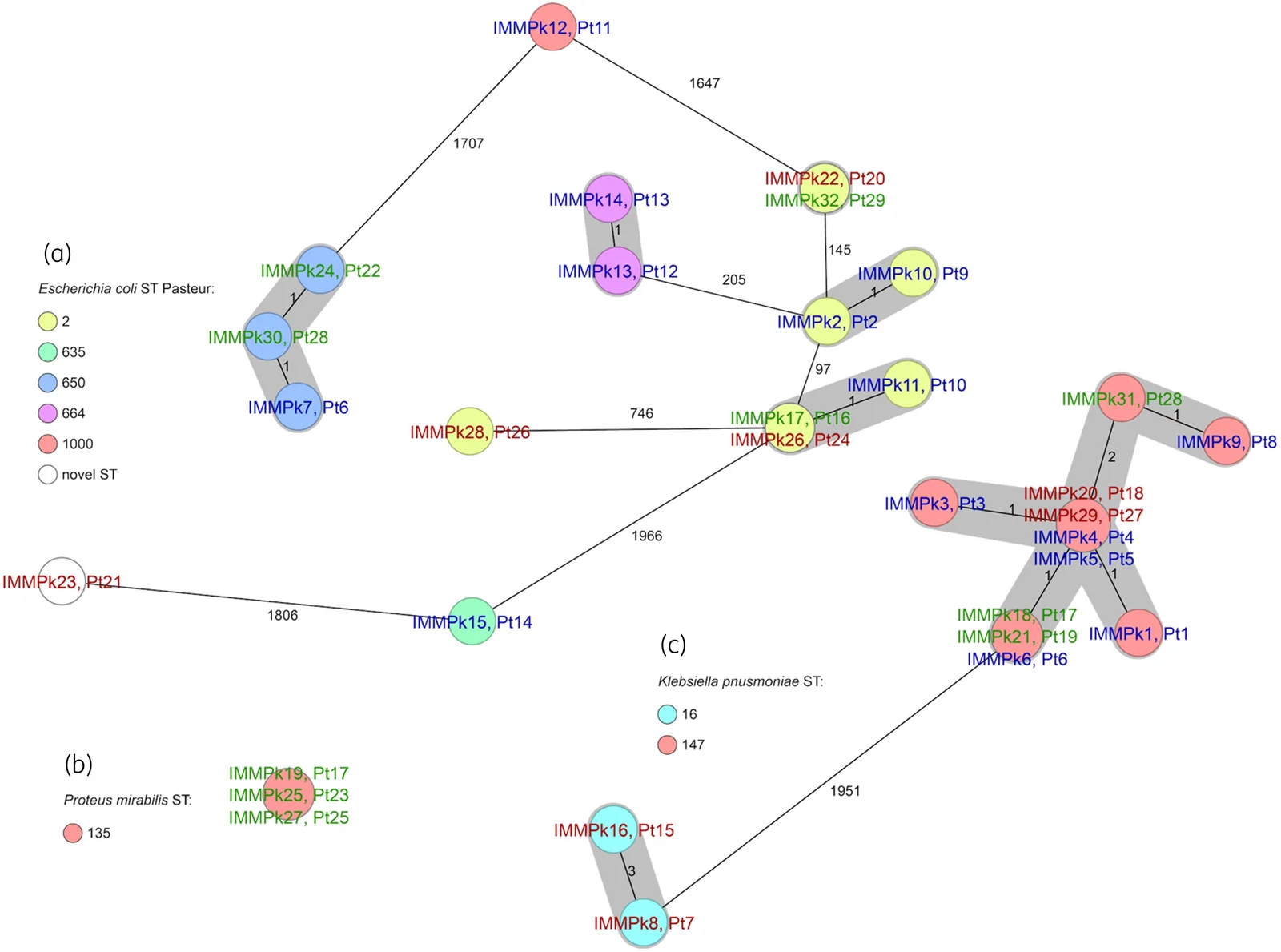
Core Genome MLST minimum spanning trees showing relatedness and ST of isolates. (a) *E. coli* (cluster threshold 10); (b) *P. mirabilis* (cluster threshold 10); (c) *K. pneumoniae* (cluster threshold 15). The relevant medical centres have been added as sample names coloured blue for the Khyber Teaching Hospital, red for the Northwest General Hospital and green for the Rehman Medical Institute.

We recovered three NDM-producing *P. mirabilis* strains from sputum and wound swabs from three patients at the Rehman Medical Institute; all belonged to ST135 (Figure 1b) and were identical by cgMLST, suggesting a likely local transmission event or a common source, although detailed epidemiological linkage data were not available.

Among the 13 *K. pneumoniae* NDM-5/OXA-232 isolates (Figure 1c), 11 belonged to ST147, forming a single overall cluster, suggesting a possible outbreak. These isolates originated from samples collected from different patients treated in three hospital centres (Khyber Teaching Hospital, Rehman Medical Institute and Northwest General Hospital). While this pattern is compatible with inter-hospital transmission, the absence of epidemiological linkage data beyond hospital origin and specimen type means that circulation of a regionally established clone in the community cannot be excluded, particularly for this ST147 lineage. All ST147 isolates were assigned as hypervirulent with a virulence score of 4 out of a maximum of 5 by Kleborate, a genotyping tool specifically designed for *K. pneumoniae* and related species that integrates detection and typing of major virulence determinants. This score reflects the presence of aerobactin and yersiniabactin but absence of colibactin (Table S1).^10^ Additionally, the *rmpA2* gene, associated with hypermucoidy, was detected in all ST147 isolates. These genomic features are consistent with a hypervirulence-associated genotype; however, phenotypic confirmation of hypervirulence, such as string testing, serum resistance assays, or *in vivo* virulence models, was not performed, and functional expression of these determinants cannot be assumed. The remaining two NDM-5/OXA-232–producing *K. pneumoniae* isolates belong to ST16 and formed a cluster differing by three cgMLST alleles. These isolates were recovered from wound swabs of two patients treated at Khyber Teaching Hospital and exhibited a virulence score of 1, corresponding to the presence of yersiniabactin only. No hypermucoidy-associated genes (*rmpA2* or the *rmpADC* locus) were detected in these isolates.

Three patients carried multiple CRE. Patients six and 28 each carried an NDM-producing *E. coli* ST650 and an NDM-5/OXA-232–producing *K. pneumoniae* ST147, in both cases recovered from wound swabs obtained in two different hospital centres (Khyber Teaching Hospital and Rehman Medical Institute). Patient 16 carried an NDM-1–producing *P. mirabilis* ST135 and an NDM-5/OXA-232–producing *K. pneumoniae* ST147.

### Phenotypic analysis

The phenotypic resistance profiles demonstrated a strong correlation with the genotypic findings. High-level resistance to third-generation cephalosporins and carbapenems was consistently observed across the isolates (Figure 2). We commonly noted elevated resistance to fluoroquinolones. Susceptibility to aminoglycosides varied among the isolates, with gentamicin retaining activity in most cases (27/32 isolates; 84%). Broad-spectrum aminoglycoside resistance mediated by rRNA methyltransferases (RMTases) was identified in 3 of 32 (9%) isolates. Among second-line antibiotics, most *E. coli* isolates exhibited low MICs for tigecycline and eravacycline, consistently remaining below the EUCAST CBP (Figure 3). Conversely, the majority of *K. pneumoniae* and *P. mirabilis* isolates showed tigecycline MICs exceeding the *E. coli* EUCAST breakpoint. However, eravacycline MICs were generally lower in these non-*E. coli* isolates and more frequently fell below the *E. coli* EUCAST CBP. All CPE isolates displayed susceptible colistin MICs. Regarding novel β-lactams, most *E. coli* isolates exhibited elevated aztreonam–avibactam MICs (4–32 mg/L). In most of these cases, the reduced susceptibility or resistance was likely resulting from coproduction of a CMY-type AmpC and/or insertions in the aztreonam target, the PBP3-encoding gene *ftsI*. The well-described YRIN insertion at amino acid 337 was present in six isolates, either alone (in two isolates with MICs of 2 mg/L) or in combination with CMY production (two CMY-145, one CMY-2), resulting in MIC values between 2 and 16 mg/L. Two additional NDM-5–producing *E. coli* contained an unusual KYRI insertion at amino acid 336; these strains coproduced CMY-2 and displayed the highest aztreonam–avibactam MICs (16–32 mg/L). In total, eight *E. coli* isolates showed aztreonam–avibactam MICs above the EUCAST clinical breakpoint. Finally, nine of 16 (56.3%) *E. coli* isolates displayed cefiderocol MICs above the EUCAST breakpoint (>2 mg/L). Eight of these nine isolates were NDM-5/OXA-48–like–producing *E. coli* belonging to ST2. Six of the nine isolates also carried an insertion in the PBP3-encoding gene *ftsI*, a mutation previously associated with reduced susceptibility to cefiderocol. All eight ST2 isolates further exhibited a frameshift mutation in codon 90 of *cirA*, resulting in premature truncation of the protein and likely contributing to the elevated MICs (4–8 mg/L). We did not detect additional known mutations in genes associated with cefiderocol resistance in isolates with higher MICs (8–32 mg/L) (Table S2).

**Figure 2.**
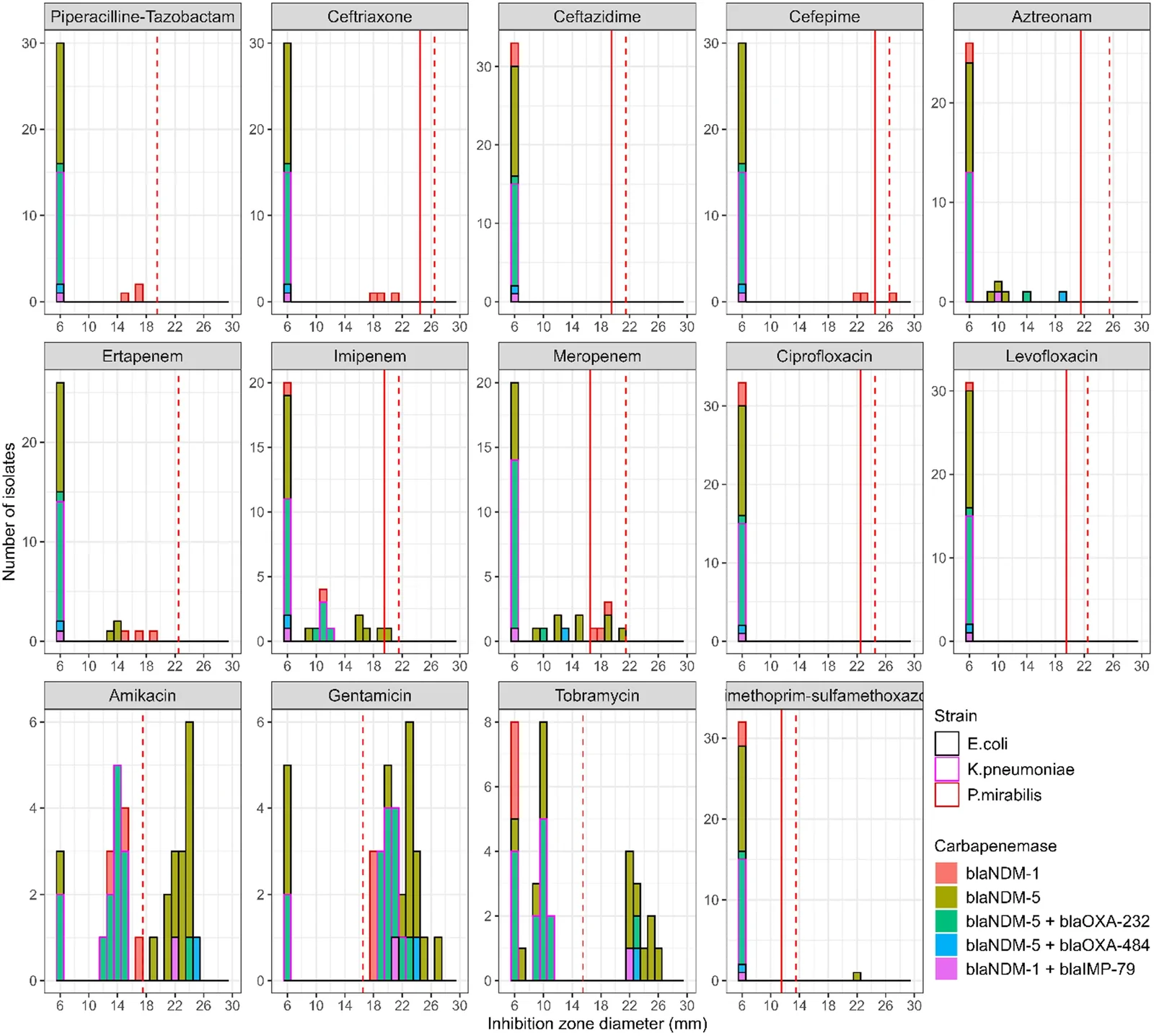
Distribution of growth inhibition zones of classic antibiotics. Vertical continuous and dashed lines denote the EUCAST resistant and susceptible clinical breakpoints, respectively.

**Figure 3.**
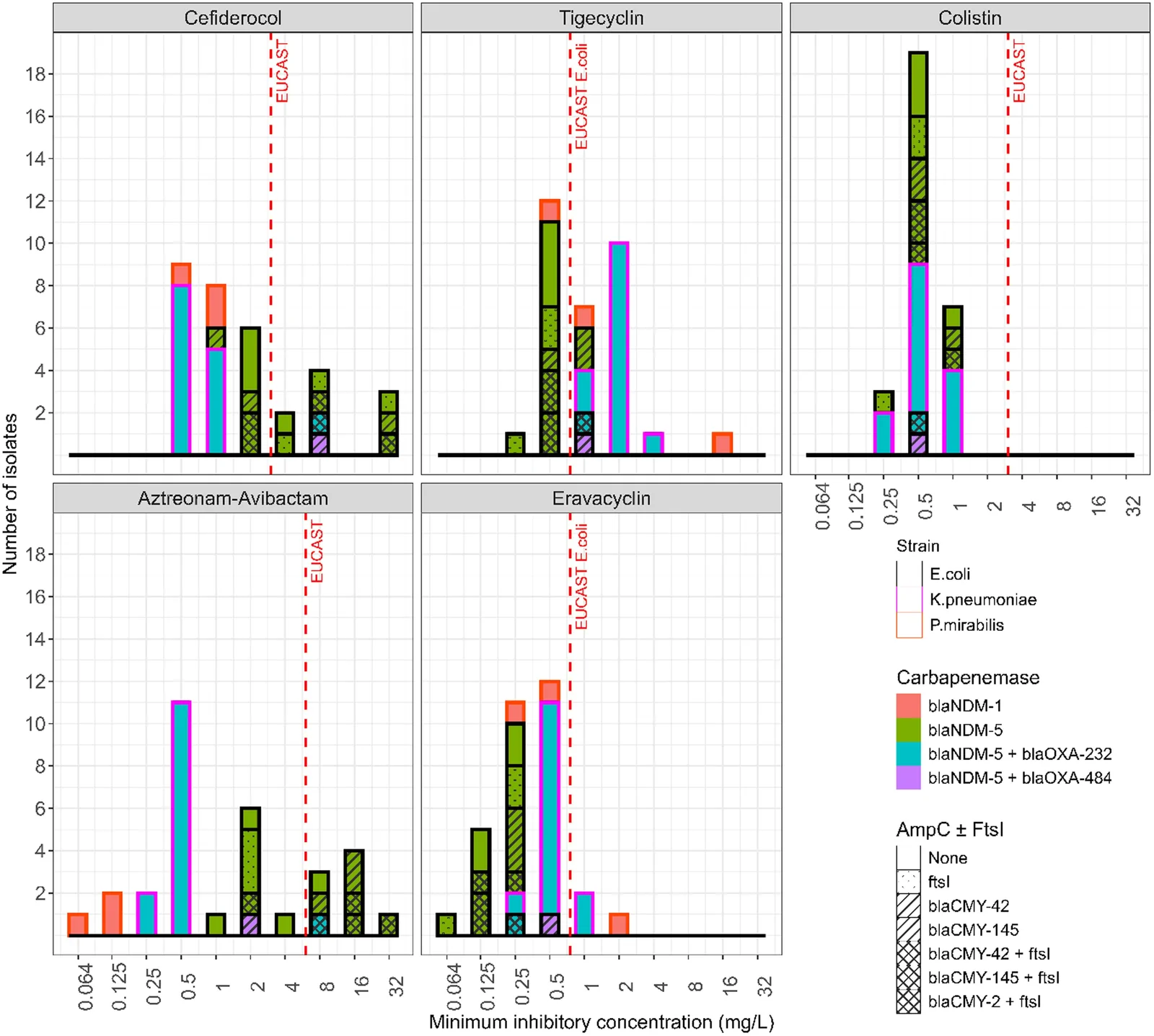
Distribution of minimum inhibitory concentrations of second-line antibiotics. Vertical dashed lines denote the EUCAST CBP.

## Discussion

Our findings confirm the sustained prevalence of NDM- and CTX-M-producers among clinical Enterobacterales isolates in northwestern Pakistan. The co-occurrence of multiple β-lactamase genes in single isolates mirrors previously reported patterns in the region, emphasizing the critical need for routine molecular surveillance.^12,13^ Consistent with earlier findings, NDM-5/OXA-232-producing *Klebsiella pneumoniae* ST147 was the predominant carbapenemase-producing lineage, further confirming the pandemic spread of this clone in Pakistan.^2^ Particularly concerning is the detection of NDM-5–producing *E. coli* belonging to several STs coproducing additional resistance mechanisms, such as the plasmid-encoded AmpC-type cephalosporinases CMY-42 and CMY-145, which are associated with reduced susceptibility or resistance to aztreonam–avibactam.^14^ Insertions in the aztreonam-binding domain of the PBP3-encoding gene *ftsI—*the main driver of aztreonam resistance and reduced susceptibility to cefiderocol through decreased β-lactam affinity for the PBP3 binding site-^15,16^ were detected in eight NDM-5–producing *E. coli*, either alone or in combination with plasmidic CMY β-lactamases. Although single-formulation aztreonam–avibactam remains unavailable or limited in Pakistan, the related β-lactam/β-lactamase inhibitor combination ceftazidime–avibactam has become accessible (since approximately 2022). In clinical practice, ceftazidime–avibactam combined with aztreonam is increasingly used as an essential regimen against MBL-producing Enterobacterales, which may promote the selection of strains with reduced susceptibility to aztreonam–avibactam. Notably, despite harbouring PBP3 insertions and/or CMY enzymes, five NDM-producing *E. coli* still showed aztreonam–avibactam MICs above the ECOFF (0.125 mg/L) yet below the EUCAST breakpoint (≤4 mg/L). The clinical relevance of these genetic determinants in isolates that remain phenotypically susceptible is currently unclear. However, clinical failures of aztreonam–avibactam therapy have been reported in infections caused by MBL-producing Enterobacterales harbouring PBP3 insertions despite exhibiting susceptible MICs. The detection of such determinants in circulating hospital isolates therefore underscores the need for close surveillance.^17^

Equally worrisome is the observation that most *E. coli* isolates showed elevated cefiderocol MICs, mostly close to the EUCAST breakpoint. This aligns with evidence that NDM-type carbapenemases can hydrolyze cefiderocol at low levels and thereby confer reduced susceptibility.^18^ Also, a *cirA* frameshift mutation leading to gene disruption—a marker typically associated with cefiderocol resistance—was also detected in all *E. coli* ST2 isolates with resistant cefiderocol MICs. PBP3 insertions, including YRIN variants, also appeared to contribute to elevated cefiderocol MICs, as all isolates carrying these mutations exceeded the breakpoint. Interestingly, two NDM-5-producing *E. coli* co-producing CMY-1 and harbouring a previously unreported *ftsI* insertion (KYRI at amino acid 336) displayed cefiderocol MICs within the susceptible range (2 mg/L), suggesting that the interaction between PBP3 mutations, β-lactamases, iron uptake and cefiderocol resistance is multifactorial and may vary depending on the specific insertion type. No additional mutations in known resistance-associated genes were identified to explain the markedly increased cefiderocol MICs (4–32 mg/L) observed in other isolates, indicating that additional, as-yet-uncharacterized factors, such as increased copy numbers or expression levels of NDM-5, may contribute to reduced susceptibility.^19^ To indirectly assess potential copy number variation, we evaluated read depth using assembly statistics and calculated the relative coverage of the corresponding contig (Table S2). Under the assumption that increased coverage may reflect higher copy number and consequently higher cefiderocol MICs, we compared relative coverage values with the observed MICs. However, the results were discordant and no clear association could be established. Therefore, we currently cannot conclude whether this observation is mechanistically relevant or contributes to cefiderocol resistance. Furthermore, because both mechanisms frequently co-occurred in the same isolates, their individual contribution to the observed MIC increase cannot be fully disentangled in the absence of isogenic mutant analysis, although a combined effect is likely. Although cefiderocol is currently not commercially available in Pakistan and no documented cases of patient treatment with imported compounds exist, the drug is gradually emerging in Pakistan, with tertiary-care centres beginning to evaluate its *in vitro* activity against multidrug-resistant Enterobacterales.^20^ Nonetheless, the circulation of strains with reduced susceptibility or resistance is particularly alarming.

Despite earlier reports of substantial colistin resistance among urine isolates of *E. coli* (19.9%) and *K. pneumoniae* (1.6%) from Peshawar,^21^ all strains in our study remained susceptible to colistin, consistent with finding from other regions of Pakistan.^2^ Although colistin remains an important last-resort treatment option for carbapenemase-producing Enterobacterales in resource-limited settings, its clinical use is limited by nephrotoxicity, narrow therapeutic window, and the lack of routine therapeutic drug monitoring in many centres. All CPE isolates also displayed MICs within the wild-type distribution for tigecycline and eravacycline (ECOFF for *K. pneumoniae*, both 2 mg/L; for *P. mirabilis*, 8 mg/L and 4 mg/L, respectively), supporting glycylcyclines as viable therapeutic alternatives. Finally, gentamicin retained activity against most CPE isolates, indicating its potential utility as a partner drug in combination regimens for severe invasive infections.

Interestingly, most *K. pneumoniae* ST147 CPE isolates also harboured genotypic markers of hypervirulence, although this sequence type has been previously associated with low virulence potential.^10,22^ The convergence of multidrug resistance and hypervirulence in these isolates represents an alarming development with important clinical and public health implications.

This study has several limitations. The short study period and limited number of isolates restrict the generalizability of the findings. Furthermore, the study design was based on a convenience sample rather than a systematic molecular epidemiological surveillance approach, and potential selection bias therefore cannot be excluded. In addition, the total number of Enterobacterales isolates processed at the participating institutions during the study period was not systematically collected, preventing reliable estimation of the overall CPE prevalence and detection rates at the institutional level. Consequently, the dataset may not fully reflect the real-world epidemiology of carbapenemase-producing Enterobacterales in the region. Extended, longitudinal surveillance programmes will therefore be essential to better characterize resistance dynamics and guide therapeutic strategies.

Taken together, these findings illustrate that although older antibiotics such as colistin, gentamicin, and partially glycylcyclines continue to provide microbiological coverage, the emergence of reduced susceptibility to advanced β-lactam agents—particularly aztreonam-avibactam and cefiderocol—necessitates strengthened, sustained surveillance and early mechanism-based detection strategies in resource-limited settings.

## Supplementary Material

dlag233_Supplementary_Data

## Supplementary data

Tables S1 and S2 are available as Supplementary data at JAC-AMR Online.
