# Supplementary material for: Molecular characterization of carbapenemase-producing Enterobacterales from Peshawar City, Khyber Pakhtunkhwa, Pakistan
Source: JAC Antimicrob Resist. 2026 Oct 23;8(5):dlag233. doi: 10.1093/jacamr/dlag233 (PMC13691608; doi:10.1093/jacamr/dlag233)
Supplement: Supplementary file 1 [file dlag233_supplementary_data.docx]

**Supplementary data**

**Table S1. Origin, clinical data, genetic features and second-line antibiotic MICs of the CPE isolates included in this study.**

| **Strain no.** | **Collection date** | **MLST** | | **Species** | **Carbapenemase(s)** | **AmpC** | **ESBL** | **Other β-lactamases** | **Porin defects** | **PBP3 mutations** | **Aminoglycoside resistance genes** | **Virulence score** | **Site of isolation** | **Hospital** | **Patient no.** | **Type of infection** | **Gender** | **MIC (µg/ml)** | | | | |
| --- | --- | --- | --- | --- | --- | --- | --- | --- | --- | --- | --- | --- | --- | --- | --- | --- | --- | --- | --- | --- | --- | --- |
|  |  | **ST Pasteur** | **ST Kleb / Proteus** |  |  |  |  |  |  |  |  |  |  |  |  |  |  | **TIG** | **ERA** | **ATM/ AVI** | **COL** | **CFD** |
| IMMPk1 | 31.07.2023 |  | 147 | *K.p.* | *bla*_NDM-5_, *bla*_OXA-232_ |  | *bla*_CTX-M-15_ |  | *omp*K36_D135DGD |  | aac(6')-Ib, aph(3')-VI | 4 | urine | Khyber Teaching Hospital | 1 | urinary tract infection | female | 2 | 0.38 | 0.38 | 1 | 1 |
| IMMPk2 | 31.07.2023 | 2 |  | *E.c.* | *bla*_NDM-5_ |  | *bla*_CTX-M-15_ | *bla*_OXA-1_ |  | *ftsI*_N337NYRIN | aac(6')-Ib-cr5 |  | urine | Khyber Teaching Hospital | 2 | urinary tract infection | male | 0.5 | 0.19 | 1.5 | 0.38 | 8 |
| IMMPk3 | 09.08.2023 |  | 147 | *K.p.* | *bla*_NDM-5_, *bla*_OXA-232_ |  | *bla*_CTX-M-15_ |  | *omp*K36_D135DGD |  | aac(6')-Ib, aph(3')-VI | 4 | Pus Swab | Khyber Teaching Hospital | 3 | otitis media | male | 2 | 0.5 | 0.5 | 0.25 | 0.5 |
| IMMPk4 | 09.08.2023 |  | 147 | *K.p.* | *bla*_NDM-5_, *bla*_OXA-232_ |  | *bla*_CTX-M-15_ |  | *omp*K36_D135DGD |  | aac(6')-Ib, aph(3')-VI | 4 | Urine | Khyber Teaching Hospital | 4 | urinary tract infection | female | 1.5 | 0.38 | 0.38 | 0.5 | 0.5 |
| IMMPk5 | 16.08.2023 |  | 147 | *K.p.* | *bla*_NDM-5_, *bla*_OXA-232_ |  | *bla*_CTX-M-15_ | *bla*_OXA-9,_ *bla*_TEM-1_ | *omp*K36_D135DGD |  | aac(6')-Ib, aph(3')-VI | 4 | Pus Swab | Khyber Teaching Hospital | 5 | sepsis | male | 2 | 0.75 | 0.5 | 0.25 | 0.5 |
| IMMPk6 | 22.08.2023 |  | 147 | *K.p.* | *bla*_NDM-5_, *bla*_OXA-232_ |  | *bla*_CTX-M-15_ |  | *omp*K36_D135DGD |  | aac(6')-Ib, aph(3')-VI | 4 | wound swab | Khyber Teaching Hospital | 6 | surgical site infection | female | 2 | 0.5 | 0.5 | 0.38 | 1 |
| IMMPk7 | 19.10.2023 | 650 |  | *E.c.* | *bla*_NDM-5_ |  | *bla*_CTX-M-15_ | *bla*_TEM-1_ |  |  |  |  | wound swab | Khyber Teaching Hospital | 6 | surgical site infection | female | 0.38 | 0.19 | 3 | 0.38 | 2 |
| IMMPk8 | 12.12.2023 |  | 16 | *K.p.* | *bla*_NDM-5_, *bla*_OXA-232_ |  | *bla*_CTX-M-15_ |  | *omp*K36_D135DD |  | aac(6')-Ib, *rmtB1* | 1 | wound swab | Northwest General Hospital | 7 | wound site infection | female | 1.5 | 0.38 | 0.5 | 0.38 | 0.5 |
| IMMPk9 | 22.08.2023 |  | 147 | *K.p.* | *bla*_NDM-5_, *bla*_OXA-232_ |  | *bla*_CTX-M-15_ |  | *omp*K36_D135DGD |  | aac(6')-Ib, aph(3')-VI | 4 | wound swab | Khyber Teaching Hospital | 8 | surgical site infection | female | 3 | 0.75 | 0.5 | 0.5 | 1 |
| IMMPk10 | 12.09.2023 | 2 |  | *E.c.* | *bla*_NDM-5_ |  | *bla*_CTX-M-15_ |  |  | *ftsI*_N337NYRIN |  |  | Urine | Khyber Teaching Hospital | 9 | urinary tract infection | female | 0.5 | 0.19 | 1.5 | 0.25 | 32 |
| IMMPk11 | 13.10.2023 | 2 |  | *E.c.* | *bla*_NDM-5_ | *bla*_CMY-145_ |  | *bla*_TEM-1_ |  | *ftsI*_N337NYRIN |  |  | wound swab | Khyber Teaching Hospital | 10 | skin and soft tissue infection | male | 0.5 | 0.19 | 16 | 0.5 | 32 |
| IMMPk12 | 19.10.2023 | 1000 |  | *E.c.* | *bla*_NDM-5_ |  | *bla*_CTX-M-15_ |  |  |  |  |  | Pus Swab | Khyber Teaching Hospital | 11 | surgical site infection | male | 0.38 | 0.19 | 8 | 0.5 | 2 |
| IMMPk13 | 23.10.2023 | 664 |  | *E.c.* | *bla*_NDM-5_ | *bla*_CMY-2_ | *bla*_CTX-M-15_ |  |  | *ftsI*_I336IKYRI | aac(3)-Iie, aac(6')-Ib-cr5 |  | pus swab | Khyber Teaching Hospital | 12 | skin and soft tissue infection | female | 0.38 | 0.125 | 24 | 0.5 | 2 |
| IMMPk14 | 23.10.2023 | 664 |  | *E.c.* | *bla*_NDM-5_ | *bla*_CMY-2_ | *bla*_CTX-M-15_ |  |  | *ftsI*_I336IKYRI | aac(3)-Iie, aac(6')-Ib-cr5 |  | Pus Swab | Khyber Teaching Hospital | 13 | foot ulcer | female | 0.5 | 0.125 | 16 | 1 | 2 |
| IMMPk15 | 27.10.2023 | 635 |  | *E.c.* | *bla*_NDM-5_ |  | *bla*_CTX-M-15_ | *bla*_OXA-10_ |  | *ftsI*_N337NYRIN | aac(3)-Iie, aac(6')-Ib-cr5, *armA* |  | wound Swab | Khyber Teaching Hospital | 14 | wound site infection | male | 0.25 | 0.064 | 1.5 | 0.5 | 4 |
| IMMPk16 | 03.12.2023 |  | 16 | *K.p.* | *bla*_NDM-5_, *bla*_OXA-232_ |  | *bla*_CTX-M-15_ | *bla*_OXA-9_ | *omp*K36_D135DD |  | *aac(6')-Ib, rmtB1* | 1 | wound swab | Northwest General Hospital | 15 | wound site infection | male | 0.75 | 0.25 | 0.19 | 1 | 0.5 |
| IMMPk17 | 03.12.2023 | 2 |  | *E.c.* | *bla*_NDM-5_ | *bla*_CMY-145_ |  | *bla*_TEM-1_ |  | *ftsI*_N337NYRIN |  |  | wound swab | Rehman Medical Institute | 16 | wound site infection | male | 0.5 | 0.125 | 2 | 0.5 | 8 |
| IMMPk18 | 04.12.2023 |  | 147 | *K.p.* | *bla*_NDM-5_, *bla*_OXA-232_ |  | *bla*_CTX-M-15_ |  | *omp*K36_D135DGD |  | aac(6')-Ib, aph(3')-VI | 4 | Sputum | Rehman Medical Institute | 17 | throat infection | male | 1.5 | 0.38 | 0.5 | 0.5 | 0.5 |
| IMMPk19 | 04.12.2023 |  | 135 | *P.m.* | *bla*_NDM-1_ |  |  |  |  |  | aac(6')-Ib3 |  | Sputum | Rehman Medical Institute | 17 | throat infection | male | 0.5 | 0.19 | 0.094 |  | 0.5 |
| IMMPk20 | 11.12.2023 |  | 147 | *K.p.* | *bla*_NDM-5_, *bla*_OXA-232_ |  | *bla*_CTX-M-15_ |  | *omp*K36_D135DGD |  | aac(6')-Ib, aph(3')-VI | 4 | Sputum | Northwest General Hospital | 18 | pneumonia | male | 1.5 | 0.38 | 0.25 | 0.5 | 1 |
| IMMPk21 | 14.12.2023 |  | 147 | *K.p.* | *bla*_NDM-5_, *bla*_OXA-232_ |  | *bla*_CTX-M-15_ | *bla*_OXA-9_ |  |  | aac(6')-Ib, aph(3')-VI | 4 | Blood | Rehman Medical Institute | 19 | gastrointestinal infection | female | 1.5 | 0.38 | 0.38 | 1 | 0.5 |
| IMMPk22 | 15.12.2023 | 2 |  | *E.c.* | *bla*_NDM-5_, *bla*_OXA-484_ | *bla*_CMY-42_ |  |  |  |  |  |  | Urine | Northwest General Hospital | 20 | urinary tract infection | female | 0.75 | 0.38 | 2 | 0.5 | 8 |
| IMMPk23 | 18.12.2023 |  |  | *E.c.* | *bla*_NDM-5_ |  | *bla*_CTX-M-15_ | *bla*_TEM-1_ |  |  |  |  | Blood | Northwest General Hospital | 21 | gastrointestinal infection | female | 0.5 | 0.125 | 2 | 0.5 | 2 |
| IMMPk24 | 19.12.2023 | 650 |  | *E.c.* | *bla*_NDM-5_ | *bla*_CMY-145_ |  |  |  |  |  |  | wound swab | Rehman Medical Institute | 22 | wound site infection | male | 0.75 | 0.25 | 12 | 1 | 2 |
| IMMPk25 | 19.12.2023 |  | 135 | *P.m.* | *bla*_NDM-1_ |  |  | *bla*_OXA-10_ |  |  | *aac*(6')-Ib-AKT |  | Sputum | Rehman Medical Institute | 23 | COPD | female | 12 | 1.5 | 0.094 |  |  |
| IMMPk26 | 20.12.2023 | 2 |  | *E.c.* | *bla*_NDM-5_ | *bla*_CMY-145_ |  | *bla*_TEM-1_ |  |  |  |  | wound swab | Northwest General Hospital | 24 | surgical site infection | female | 0.5 | 0.19 | 12 | 0.5 | 32 |
| IMMPk27 | 21.12.2023 |  | 135 | *P.m.* | *bla*_NDM-1_ |  |  |  |  |  | aac(6')-Ib3 |  | wound swab | Rehman Medical Institute | 25 | surgical site infection | male | 1 | 0.32 | 0.064 |  | 1 |
| IMMPk28 | 25.12.2023 | 2 |  | *E.c.* | *bla*_NDM-5_ |  | *bla*_CTX-M-15_ |  |  |  | *aac*(6')-Ib-D181Y |  | wound swab | Northwest General Hospital | 26 | wound site infection | male | 0.38 | 0.125 | 1 | 1 | 4 |
| IMMPk29 | 25.12.2023 |  | 147 | *K.p.* | *bla*_NDM-5_, *bla*_OXA-232_ |  | *bla*_CTX-M-15_ | *bla*_OXA-9_ |  |  | *aac*(6')-Ib-AKT | 4 | wound swab | Northwest General Hospital | 27 | wound site infection | male | 1 | 0.32 | 0.38 | 1 | 1 |
| IMMPk30 | 26.12.2023 | 650 |  | *E.c.* | *bla*_NDM-5_ | *bla*_CMY-145_ |  |  |  |  |  |  | wound swab | Rehman Medical Institute | 28 | wound site infection | male | 0.75 | 0.19 | 8 | 0.5 | 1 |
| IMMPk31 | 26.12.2023 |  | 147 | *K.p.* | *bla*_NDM-5_, *bla*_OXA-232_ |  | *bla*_CTX-M-15_ |  | *omp*K36_D135DGD |  | aac(6')-Ib, aph(3')-VI | 4 | wound swab | Rehman Medical Institute | 28 | wound site infection | male | 2 | 0.5 | 0.5 | 0.5 | 0.5 |
| IMMPk32 | 26.12.2023 | 2 |  | *E.c.* | *bla*_NDM-5_, *bla*_OXA-232_ | *bla*_CMY-42_ |  |  |  | *ftsI*_N337NYRIN |  |  | wound swab | Rehman Medical Institute | 29 | wound site infection | female | 0.75 | 0.25 | 6 | 0.5 | 8 |

*K.p., K. pneumoniae; E.c., E. coli; P.m., P. mirabilis*; TIG, tigecycline; ERA, eravacycline; ATM/AVI, aztreonam-avibactam; COL, colistin, CFD, cefiderocol; virulence score 1 = yersiniabactin only, 4 = aerobactin with yersiniabactin (no colibactin). ^1^

**Table S2. Genes potentially associated with cefiderocol resistance in Enterobacterales isolates.**

| **Strain no.** | **Strain** | **ST (Pasteur)** | **Carbapenemase(s)** | **Cefiderocol MIC (mg/L)** | ***cirA*** | **NDM contig relative read depth*** | ***efeO*** | ***exbB*** | ***exbD*** | ***fecA*** | ***fiu*** | ***tonB*** | **PBP3 mutations** |
| --- | --- | --- | --- | --- | --- | --- | --- | --- | --- | --- | --- | --- | --- |
| IMMPk28 | *E.coli* | 2 | blaNDM-5 | 4 | frameshift at codon 90 | 1.05 |  |  |  |  |  |  | *-* |
| IMMPk2 | *E.coli* | 2 | blaNDM-5 | 8 | frameshift at codon 90 | 0.98 |  |  |  | V444L |  |  | *ftsI*_N337NYRIN |
| IMMPk17 | *E.coli* | 2 | blaNDM-5 | 8 | frameshift at codon 90 | 1.08 |  |  |  | V444L |  |  | *ftsI*_N337NYRIN |
| IMMPk22 | *E.coli* | 2 | blaNDM-5, blaOXA-484 | 8 | frameshift at codon 90 | 1.28 |  |  |  |  |  |  | - |
| IMMPk32 | *E.coli* | 2 | blaNDM-5, blaOXA-232 | 8 | frameshift at codon 90 | 1.28 |  |  |  |  |  |  | *ftsI*_N337NYRIN |
| IMMPk10 | *E.coli* | 2 | blaNDM-5 | 32 | frameshift at codon 90 | 1.02 |  |  |  |  |  |  | *ftsI*_N337NYRIN |
| IMMPk11 | *E.coli* | 2 | blaNDM-5 | 32 | frameshift at codon 90 | 1.06 |  |  |  |  |  |  | *ftsI*_N337NYRIN |
| IMMPk26 | *E.coli* | 2 | blaNDM-5 | 32 | frameshift at codon 90 | 1.85 |  |  |  |  |  |  | - |
| IMMPk14 | *E.coli* | 2 | blaNDM-5 | 4 | T316A | 1.37 |  |  |  | A190T | G224R, V239I, T493A, M513V | A51T, I114V | *ftsI*_N337NYRIN |

*In these isolates, *bla*_NDM-5_ is located on a contig of 8-10kb. The relative read depth of this contig in these genomes is taken from the assembly statistics.

**Reference list**

1.	Lam MMC, Wick RR, Watts SC et al. A genomic surveillance framework and genotyping tool for *Klebsiella pneumoniae* and its related species complex. *Nat Commun* 2021; **12**: 4188.
